# Hypometabolism and atrophy patterns associated with Niemann-Pick type C

**DOI:** 10.1186/s13550-025-01208-8

**Published:** 2025-02-26

**Authors:** Jesús Silva-Rodríguez, Cristina Castro, Julia Cortés, Manuel Arias, Virginia Pubul, Alexis Moscoso, Michel J. Grothe, Gabriel Reynes-Llompart, Laura Rodríguez-Bel, Jordi Gascon-Bayarri, María Jesús Sobrido, Pablo Aguiar

**Affiliations:** 1 Reina Sofia Alzheimer Centre, CIEN Foundation, ISCIII, Madrid, Spain; 2 Centro de Investigación Biomédica en Red sobre Enfermedades Neurodegenerativas (CIBERNED), Instituto de Salud Carlos III, Madrid, Spain; 3 Centro de Investigación Biomédica en Red de Enfermedades Raras (CIBERER), Instituto de Salud Carlos III, Madrid, Spain; 4 Nuclear Medicine Department and Molecular Imaging Group, University Hospital of Santiago de Compostela, IDIS, Travesía da Choupana s/n, Santiago de Compostela, Spain; 5 Neurology Department, University Hospital of Santiago de Compostela, Galicia, Spain; 6 Wallenberg Centre for Molecular and Translational Medicine, University of Gothenburg, Gothenburg, Sweden; 7 Department of Psychiatry and Neurochemistry, Institute of Physiology and Neuroscience, University of Gothenburg, Gothenburg, Sweden; 8 Department of Medical Physics, Hospital Universitari de Bellvitge-ICO L’Hospitalet (IDIBELL), L’Hospitalet de Llobregat, Barcelona, Spain; 9 Unidad PET IDI, Servicio de Medicina Nuclear, Hospital Universitari de Bellvitge, L’Hospitalet de Llobregat, Barcelona, Spain; 10 Neurology Department, Hospital Universitari de Bellvitge, L’Hospitalet de Llobregat, Catalonia, Spain; 11 Neurogenetics Research Group, Institute of Biomedical Research (INIBIC), University Hospital of A Coruña, Galicia, Spain; 12 Molecular Imaging Group, Center for Research in Molecular Medicine and Chronic Diseases (CIMUS), University of Santiago de Compostela (USC), Campus Vida, Santiago de Compostela, Galicia, Spain; 13 Nuclear Medicine Department, Choupana s/n, Santiago de Compostela, 15706 Spain; 14 Instituto de Investigación Biomédica de A Coruña, Xubias de Arriba, 84, A Coruña, 15006 Spain; 15 Carrer de la Feixa Llarga, s/n, Bellvitge 08907 L’Hospitalet de Llobregat, Barcelona, 08907 Spain

## Abstract

**Background:**

Niemann–Pick disease type C (NP-C) is a rare genetic lysosomal lipid storage disorder characterized by progressive neurological impairment. Early diagnosis is critical for initiating treatment with miglustat, which can decelerate disease progression. In this study, we evaluated a cohort of 22 NP-C patients who underwent MRI, [^18^F]FDG PET, and clinical assessment at baseline. We performed a cross-sectional and longitudinal imaging study evaluating the role of [^18^F]FDG PET as an adjunct diagnostic tool for NP-C alongside MRI, the current neuroimaging standard.

**Results:**

Group-level MRI analysis identified significant cerebellar and thalamic atrophy (d = 1.56, *p* < 0.0001 and d = 1.09, *p* < 0.001, respectively), with less pronounced involvement of the frontal lobe and hippocampus, which aligned with existing neuropathological understanding and guidelines. Conversely, [^18^F]FDG PET imaging revealed extensive hypometabolism in the cerebellum, thalamus, and cingulate cortex (d = 1.42, *p* < 0.0001), and moderate hypometabolism in broad frontotemporal areas. [^18^F]FDG PET provided higher effect sizes across all brain regions, including regions without apparent atrophy, which suggests that it may be more sensitive than MRI for detecting NP-C neurodegenerative changes. Single-subject visual assessment of individual PET images further validated the clinical utility of [^18^F]FDG PET, with significant hypometabolism observed in the cerebellum, thalamus and anterior and posterior cingulate reported by physicians in 17/22 patients. Both hypometabolism and atrophy in the cerebellum were associated with ataxia, (more strongly indicated by [^18^F]FDG PET, *p* < 0.0001 vs. MRI, *p* = 0.07). Medial temporal lobe atrophy was associated with cognitive impairment (*p* < 0.05), and frontal hypometabolism was slightly related to behavioural impairment (*p* < 0.07). Longitudinal [^18^F]FDG PET analysis revealed progressive subcortical, cortical and cerebellar hypometabolism, which was most pronounced in the cerebellum (-12% per year, *p* < 0.001). Patients treated with miglustat showed a trend towards attenuated cerebellar hypometabolism progression compared to untreated patients (*p* = 0.10).

**Conclusions:**

Our findings delineate a discernible hypometabolism pattern specific to NP-C that distinguishes it from other neurodegenerative conditions, thus suggesting that [^18^F]FDG PET might be a promising tool for NP-C diagnosis and to study disease progression.

**Trial registration:**

XUNTA 2015/140. Registered 21 April 2015.

**Supplementary Information:**

The online version contains supplementary material available at 10.1186/s13550-025-01208-8.

## Introduction

Niemann–Pick disease type C (NP-C) is a rare and highly debilitating lysosomal lipid storage disorder caused by mutations in NPC1 (95% of patients) or NPC2 (5% of patients) genes [1, 2]. NP-C patients exhibit progressive neurological impairment and highly variable neuropsychiatric symptoms, including cerebellar ataxia, vertical supranuclear gaze palsy, pyramidal features, dystonia, dysarthria, dysphagia, seizures, progressive hearing loss, major depression, psychosis and cognitive decline [3, 4]. The current diagnostic workup includes blood biomarkers (chitotriosidase, oxysterols, bile acids, Lyso-SM-509), NPC1/NPC2 gene sequencing, and filipin staining of unesterified cholesterol in cultured fibroblasts obtained from a skin biopsy [4]. Different combinations are often applied that may involve screening and confirmatory testing [5]. Like many rare disorders, diagnosis of NP-C is often challenging and the differential diagnosis against other conditions can be time-consuming, thus innovative biomarkers capable of confirming the presence of NPC-like neurodegeneration could play a critical role in supporting the diagnosis [6]. Moreover, early diagnosis of NP-C is paramount, since a glucosylceramide synthase inhibitor treatment, miglustat, is available to slow down progressive neurological manifestations in adults and children. In this regard, biomarkers that can track disease progression are also needed [7].

Nowadays, it is well-known that the abnormal neuronal storage of lipids precedes neurodegeneration in NP-C. This was first described in animal models, showing that Purkinje cells in the cerebellum, basal ganglia, and thalamus were first affected, followed by neurons in the hippocampus and cortical regions [8, 9]. Brain magnetic resonance imaging (MRI) confirmed these findings in humans, revealing atrophy patterns broadly consistent with the animal model neuropathology [10]. Moreover, significant cerebellar grey and white matter volume reductions in NP-C patients were found to be associated with ataxia [11]. Some patients also showed atrophy in the midbrain [12] and the corpus callosum [13]. Furthermore, diffusion tensor imaging (DTI) revealed reductions in fractional anisotropy in major white matter tracts [14–17], which is consistent with the hypothesis of disrupted myelination preceding neuronal cell body loss [18]. All these neuroimaging findings were summarized in the current recommendations for the detection and diagnosis of NP-C [19]. However, the guidelines also remark that MRI changes are detected only in the advanced stages of the disease. Thus, the absence of MRI abnormalities may not exclude a diagnosis of NP-C, and complementary imaging modalities may be needed to aid early diagnosis. In this regard, positron emission tomography (PET) with the glucose analogue [^18^F]fluorodeoxyglucose ([^18^F]FDG) as a radiotracer is a well-established imaging tool for imaging neurodegeneration, and in recent years accumulating evidence across different neurodegenerative disorders suggests that [^18^F]FDG PET functional changes may precede atrophy as measured by MRI [20–24]. Recently, Lau et al. [25] reported that NP-C might be accompanied by a distinctive [^18^F]FDG PET hypometabolism pattern characterized by frontal, thalamic, and parietal hypometabolism. In addition, previous works, mainly case reports, suggested that NP-C patients may show severe hypometabolism even in the presence of very subtle or no MRI abnormalities [26–28]. These findings still need confirmation in larger patient cohorts.

Here, we conducted a cross-sectional and longitudinal [^18^F]FDG PET and MRI study in a relatively large cohort of NP-C patients, in order to define whether (a) there is a characteristic hypometabolic pattern related to NP-C; (b) hypometabolism is more pronounced and easier to detect than atrophy in NP-C; (c) individual [^18^F]FDG PET patterns are useful for the clinical diagnosis of NP-C; and (d) if [^18^F]FDG PET is able to measure longitudinal changes in NP-C, allowing to monitor progression and treatment response.

## Materials and methods

### Patient recruitment and clinical evaluation

This is a national, multicentre, observational, longitudinal cohort study. The protocol for the study was approved by the Galician Research Ethics Committee (XUNTA 2015/140). The aims and study protocol were disseminated through the Spanish scientific networks for rare and metabolic diseases, the Spanish committee on ataxias - movement disorders working group, the Spanish Society of Neurology, and different patient associations (rare disease, metabolic diseases) to recruit patients fulfilling recruitment criteria and willing to participate. Eligible subjects must have a genetically confirmed diagnosis of NP-C disease (any combination of clinical manifestations was permitted) or inconclusive genetic diagnosis combined with characteristic NP-C symptomatology (evaluated by a panel of neurologists specialized in NPC). Subjects with an uncertain genetic and clinical diagnosis, medical contraindication for [^18^F]FDG PET study, very severe disability due to NP-C or to other concomitant diseases (Barthel Index for Activities of Daily Living (ADL) ≤ 20) or unable to travel, were excluded. All participants signed informed consent to participate in the study.

Retrospective data collection was completed by the patient’s referring physician and the clinical collaborators of the study, and all the information was standardized to homogenize patient-specific demographic and clinical data (gender, age at the moment of data collection, age at the beginning of the disease, age at diagnosis, first symptoms, years of evolution, time of treatment, family history, observational prospective neurological evaluation, subjective evaluation, pertinent complementary tests) as well as complementary tests and examinations (genetic test, filipin staining, biochemical analysis, ultrasounds, electroencephalography, videonystagmography and disability score).

### Neuroimaging acquisition

At baseline, all the participants travelled to the nearest reference hospital the day before carrying out the neuroimaging scans to ensure similar basal conditions (i.e., avoiding travel-related differences in wakefulness and fatigue on the day of scanning). MRI and PET scans were taken early in the morning, after at least 8 h of fasting.

The MRI protocol consisted of T1-weighted 3D TFE axial high-resolution images, with TR, 8.73 milliseconds; TE, 3.96 milliseconds; section thickness, 0.8 mm; and distance, 0.4 mm. The used MR scanners were Philips Achieva (Hospital Clínico de Santiago de Compostela and Centro de Investigaciones Médico-Sanitarias, Málaga), Siemens Biograph mMR (Hospital Puerta de Hierro, Madrid), Siemens Magnetom Vida (Clínica Corachan, Barcelona) and Siemens Magnetom Essenza (Hospital de Cruces, Bilbao).

[^18^F]FDG PET acquisitions were performed 30–45 min after the intravenous injection of 185–370 MBq of [^18^F]FDG. Patients were asked to lay at rest in a dark and quiet room during the uptake period. Patients were scanned for 10–30 min using a bed covering the whole brain (special care was taken to include the whole cerebellum). The PET scanners in use were GE Advance NXi (Hospital Clínico de Santiago de Compostela), Siemens Biograph mMR (Hospital Puerta de Hierro, Madrid), GE Discovery ST (Hospital Universitari Bellvitge, Barcelona and Centro de Investigaciones Médico Sanitarias, Málaga) and GE Discovery 690 (Hospital Cruces, Bilbao).

### Image processing

Joint analysis of the multicentre structural MRI and [^18^F]FDG PET images was performed using Neurocloud (Qubiotech SL, https://www.qubiotech.com/en/solutions/), a cloud software platform providing a simple, automatic, and easy-to-use interface to most SPM12 functionalities and several of its toolboxes, including the Computational Anatomical Toolbox (CAT) [29]. The software incorporates a CE-mark for its clinical use in the European Union.

Structural MRI pre-processing was performed using standard CAT pipelines, which included the initial application of a spatial adaptive non-local means denoising filter [30], followed by an internal resampling to deal with different resolution images and anisotropic spatial resolution between scanners. The data were then bias-corrected, segmented into tissue classes, and spatially normalized using SPM’s “unified segmentation” approach [31, 32]. The output voxel size of the normalized images was 1.5 × 1.5 × 1.5 mm^3^. Segmented grey matter (GM) tissue maps were smoothed using an isotropic 10 × 10 × 10 mm^3^ kernel to remove differences related to inter-individual differences prior to statistical analysis.

PET pre-processing started with the application of scanner-specific smoothing kernels provided by Neurocloud for each scanner were applied, aiming to obtain a uniform isotropic resolution of 8 mm FWHM (Supplementary Table 1). Next, images were spatially normalized of a custom [^18^F]FDG template derived from a healthy subject database of 78 subjects previously acquired at our center [33]. Normalization included a standard twelve-affine spatial transformation followed by low-dimensional non-linear transformations [29]. The output voxel size of the normalized images was 1.5 × 1.5 × 1.5 mm^3^. Finally, a histogram-based intensity normalization method was applied [34, 35]. In brief, we calculated voxel-wise normalization maps by dividing the [18F]FDG template by the input image. Then, the most prevalent value in the derived normalization map (i.e. the histogram peak) is selected as the global normalization factor to scale the input image [34]. After preprocessing, resulting PET images were smoothed with an additional 6 × 6 × 6 mm^3^ kernel prior to statistical analysis, resulting in a final intrinsic resolution of approximately 10 mm in every direction.Regional values of grey matter volume (GM_VOL, MRI) and standardized uptake value ratios (SUVR, [^18^F]FDG PET) were derived from the pre-processed images by using the volumes of interest (VOIs) provided by the Hammersmith atlas [36].

### Statistical analysis

#### Group-level analyses

Region-based and voxel-based analysis were performed by comparing NP-C patients against a reference group of age-matched healthy subjects. Healthy subjects underwent the same preprocessing used for NP-C patient images.

For structural MRI, the reference group included a sample of 100 age-matched subjects from the IXI (brain-development.org) and OASIS (oasis-brains.org) databases, automatically selected by Neurocloud to best match the patient’s age. Grey matter voxel-based morphometry (VBM) analysis was conducted as implemented in CAT12 [31]. Age at MRI acquisition, gender and total intracranial vme (TIV) were used as confounding nuisance covariates. The analysis was constraint to the interior of a generic parenchyma mask provided by CAT12. Statistical differences were transformed to z-scores and overlapped over rendered brain and cerebellum templates after applying a statistical threshold of z-score = ± 1.5. A minimum cluster size of 250 voxels was also applied. Only reductions in volume were considered, while volume increases were only visually inspected to identify any potential artifacts. After statistical analysis, z-scores were transformed into Cohen’s d to provide a measure of effect size in visual presentations.

For [^18^F]FDG PET, the reference group was the aforementioned control database of 78 control subjects (Age: 59 ± 14), acquired at our centre using the same image acquisition protocol used for the NP-C group [33]. Briefly, subjects were scanned in a GE Advance NXi for 30 min, starting 45 min after the injection of 370 MBq of [^18^F]FDG. Images were reconstructed with an iterative reconstruction method using 4 iterations and 16 subsets. Voxel-wise analysis was performed by using the two-sample t-test statistical module in SPM12 [29], constraint to the interior of the same parenchyma mask used for VBM analysis. Age at PET acquisition and sex were used as confounding nuisance covariates. As for MRI analysis, statistical differences were transformed to z-scores and projected onto brain and cerebellum templates after applying a threshold of z-score = ± 1.5 and a minimum cluster size of 250 voxels. As for VBM maps, z-scores were transformed into Cohen’s d to represent effect size.

In addition to voxel-based comparisons, we performed group-wise region-based analysis for both modalities using the previously derived GM_VOL and SUVR values. Comparisons between the NP-C group and the corresponding age-matched healthy control group were performed for each region by using two-sample t-tests. Statistical differences were overlapped over a rendered brain template. Additionally, we also performed two-sample t-tests to assess differences in GM_VOL and SUVR between several clinically relevant pathological subgroups, namely patients with and without ataxia, with and without cognitive or behavioural impairment, or with and without long-term miglustat treatment. Differences are reported as effect size (Cohen’s d) and statistical significance. Differences were considered significant at a *p* < 0.01 (uncorrected). Finally, Pearson correlations were performed to assess the associations between GM_VOL and SUVR regional values and patient’s age and years since the clinical onset.

We also evaluated the correlation between hypometabolism and atrophy patterns and relevant clinical symptoms, including ataxia, cognitive impairment, and behavioural impairment. To this end, we used two-sample t-tests. Results are reported as effect size (Cohen’s d) and statistical significance. The correlation of atrophy and hypometabolism between each other, and with other relevant variables such as age and years from onset was assessed using the Pearson’s r test. For these analyses we used *p* < 0.05 (uncorrected) as an statistical threshold.

#### Single-subject analyses

Considering the clinical heterogeneity of NP-C, the usefulness of the obtained hypometabolism patterns in the context of individual patient diagnosis was evaluated in complementary single-subject analyses. First, single-subject voxel-wise comparisons between each NP-C patient and the corresponding healthy subject database were performed by using the single-subject analysis pipelines incorporated in Neurocloud, which implement an approach based on Statistical non-parametric mapping (SnPM, http://www.nisox.org/Software/SnPM13/). Next, two experienced nuclear medicine physicians performed a consensual visual rating (without quantitative information) of each subject’s raw PET image, assigning a score of 0 (none), 1 (mild), 2 (moderate), or 3 (severe) hypometabolism to frontal, temporal, parietal, occipital and cingulate cortical regions, thalamus, basal ganglia, and cerebellum. Physicians were also asked to score between 0 (none), 1 (average), 2 (good) and 3 (very good) the correlation between the observed visual abnormalities and the group-level hypometabolism pattern, which was freely available to them during the visual assessment.

#### Longitudinal analysis

For longitudinal analysis, the individual percentage change in regional metabolism (%/year) was calculated based on the region-based analysis (using the deviation from the average healthy subject’s metabolism as a metric). The significance of the calculated changes was assessed by applying one-sample t-tests comparing the measured change to zero. Furthermore, differences in hypometabolism progression between patients with and without ataxia, cognitive impairment, or long-term miglustat treatment were assessed using linear mixed effects models, which included patient-specific intercepts and slopes. Sex and age were used as nuisance covariates for all models.

## Results

### Patient recruitment

Twenty-two patients with genetically confirmed NP-C (mean age 39.6 ± 13.7, 9 females) were recruited along different Neurology Departments in Spain. Demographic characteristics and details of the neuroimaging acquisitions are summarized in Supplementary Table 2. Neuroimaging protocols were fulfilled in most patients. In a small number of patients, the MRI study was delayed for several months due to scheduling or patient disability problems, and it could not be performed on three patients. Neuroimaging acquisition was performed at varying intervals after clinical onset (13.3 ± 9.3 years). Twelve patients were able to complete at least one follow-up visit. The mean follow-up time was 38.1 ± 40.6 months and was highly dependent on the patient’s distance from the reference center and the clinical evolution.

**Fig. 1:**
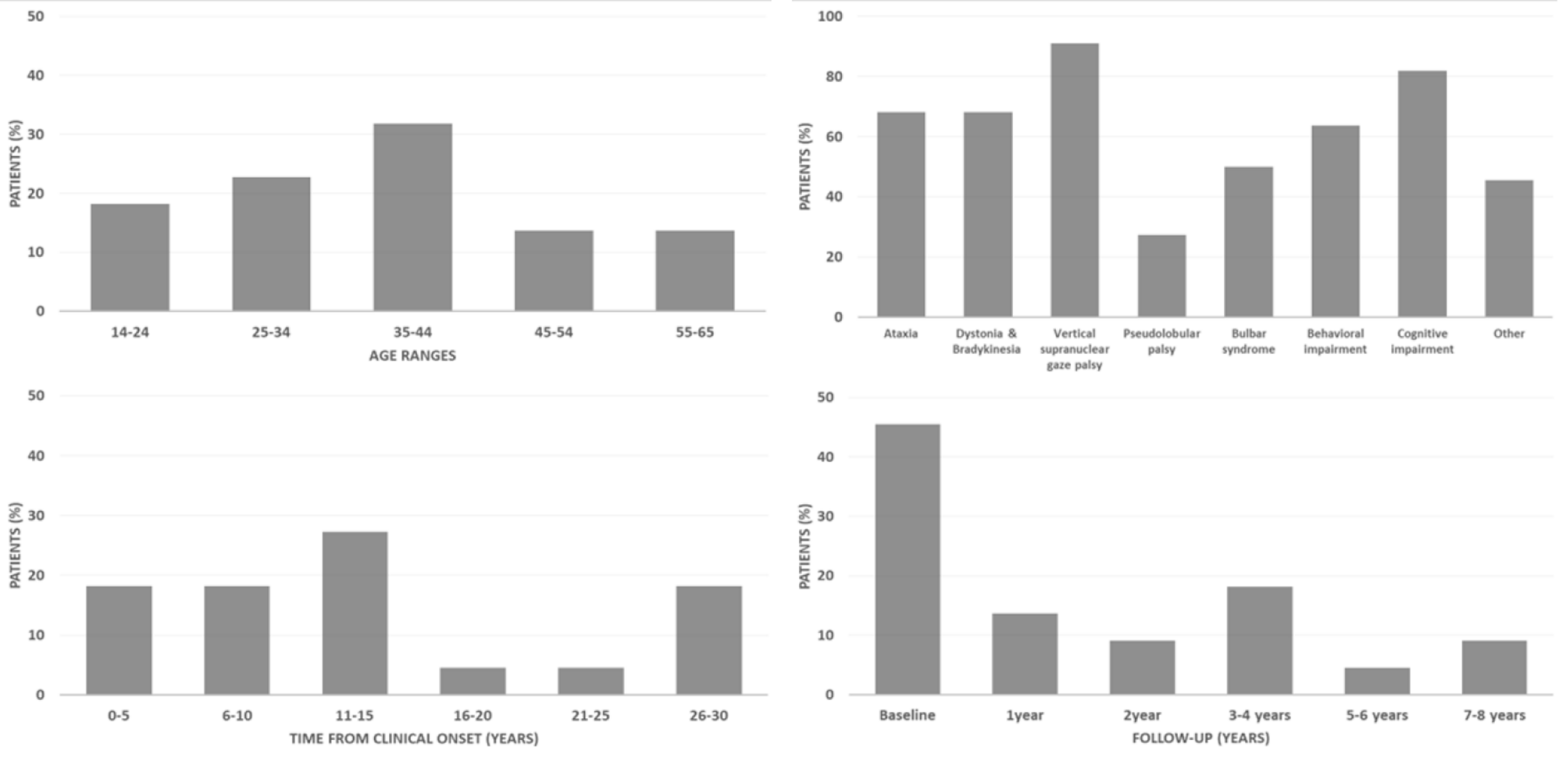
Number of patients (% of total) in terms of age range, clinical symptoms, time from clinical onset, follow-up time

Figure 1 summarizes general characteristics along the cohort and Supplementary Table 3 shows patient-specific information. Neurological assessment was performed at the same day as the baseline neuroimaging studies as well as the detected NPC mutations. In terms of clinical evaluation, cognitive impairment and ataxia were the most common symptoms throughout the recruited cohort. Ataxia was present in 15/22 patients (68%), while cognitive impairment was present in 18/22 patients (82%), of which 14/18 (77%) also presented behavioural impairment. In terms of NPC gene variants, all mutations were found in NPC1, except patient P0003, who had a mutation in NPC2. Note that no NPC mutation was found for P0019. Half of the patients (11/22) were under treatment with miglustat.

### Baseline group-level analysis

**Fig. 2:**
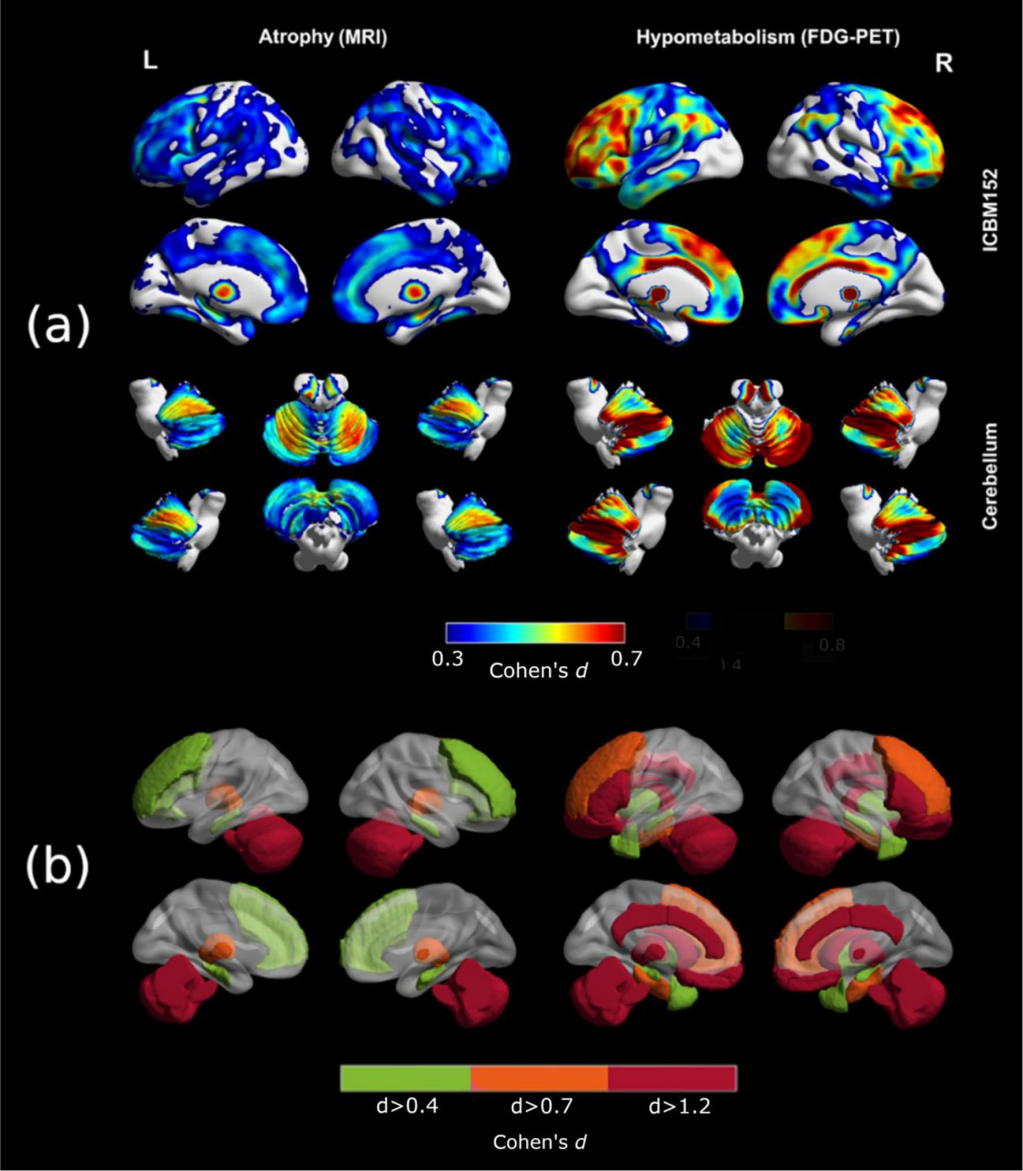
Averaged patterns of atrophy and hypometabolism derived from group-level analysis

Figure 2 (a) shows the voxel-wise spatial patterns of atrophy and glucose hypometabolism across all NP-C patients. MRI analysis revealed significant atrophy in the cerebellum, thalamus and hippocampus, with additional mild involvement of cortical regions, especially in the frontal lobe. By contrast, the hypometabolism pattern extends throughout the entire cerebellum and limbic system, affecting the thalamus, midbrain, basal ganglia, anterior and posterior cingulate cortex, and large frontal, parietal and temporal bilateral cortical regions. No asymmetries were found, neither in atrophy nor in hypometabolism patterns.

Figure 2 (b) presents the results of the complementary region-based analysis, which largely confirms the voxel-wise results. Significant volume reductions compared to the healthy subjects were found in the cerebellum (d = 1.56, *p* < 0.0001) and thalamus (d = 1.09, *p* < 0.001), as well as middle frontal gyrus (d = 0.56, *p* < 0.01) and hippocampus (d = 0.61, *p* < 0.01). On the other hand, ^[18 F]^FDG uptake was significantly decreased in the cerebellum (d = 1.42, *p* < 0.0001), thalamus (d = 1.42, *p* < 0.0001), anterior and posterior cingulate (d > 1.4, *p* < 0.0001), as well as in the caudate nucleus, straight gyrus, and orbitofrontal gyrus (d > 1.2, *p* < 0.0001); middle frontal gyrus and parahippocampus (d > 0.73, *p* < 0.001); and hippocampus, anterior temporal lobe and putamen (d > 0.44, *p* < 0.01). As it might be expected, we observed a significant positive correlation between atrophy and hypometabolism on several areas, such as the cerebellum, thalamus, posterior cingulate, fusiform gyrus, and middle and inferior frontal gyrus (*r* > 0.46, *p* < 0.05). However, we did not find statistically significant correlations between regional hypometabolism (or atrophy) and the patient’s age (-0.37 < *r* < 0.33) or years since the clinical onset (-0.31 < *r* < 0.42).

### Single-subject analysis

**Fig. 3:**
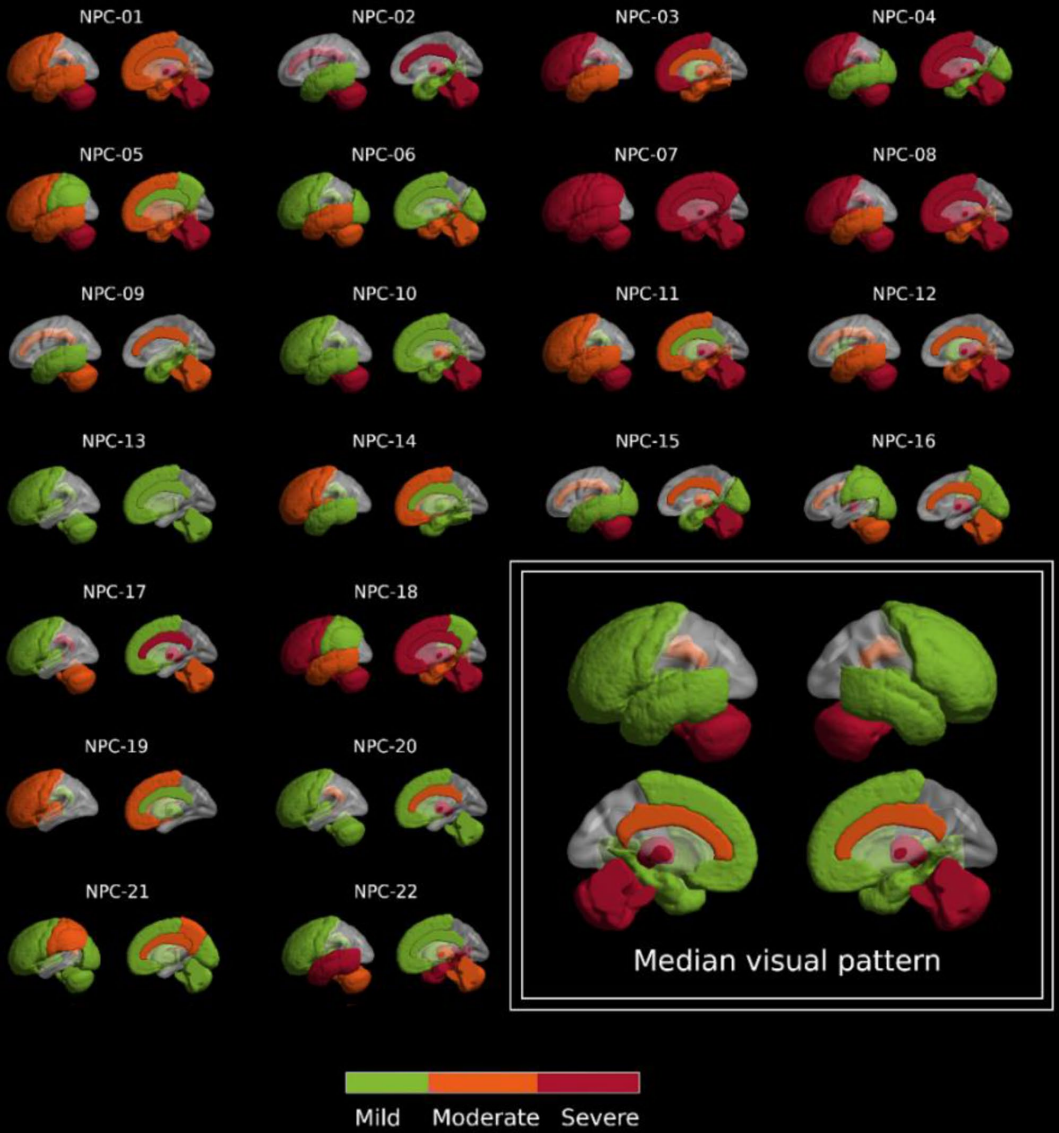
Described visual pattern for each of the 22 NP-C patients. In the left-bottom corner, the median of each ROI over the whole database

**Fig. 4:**
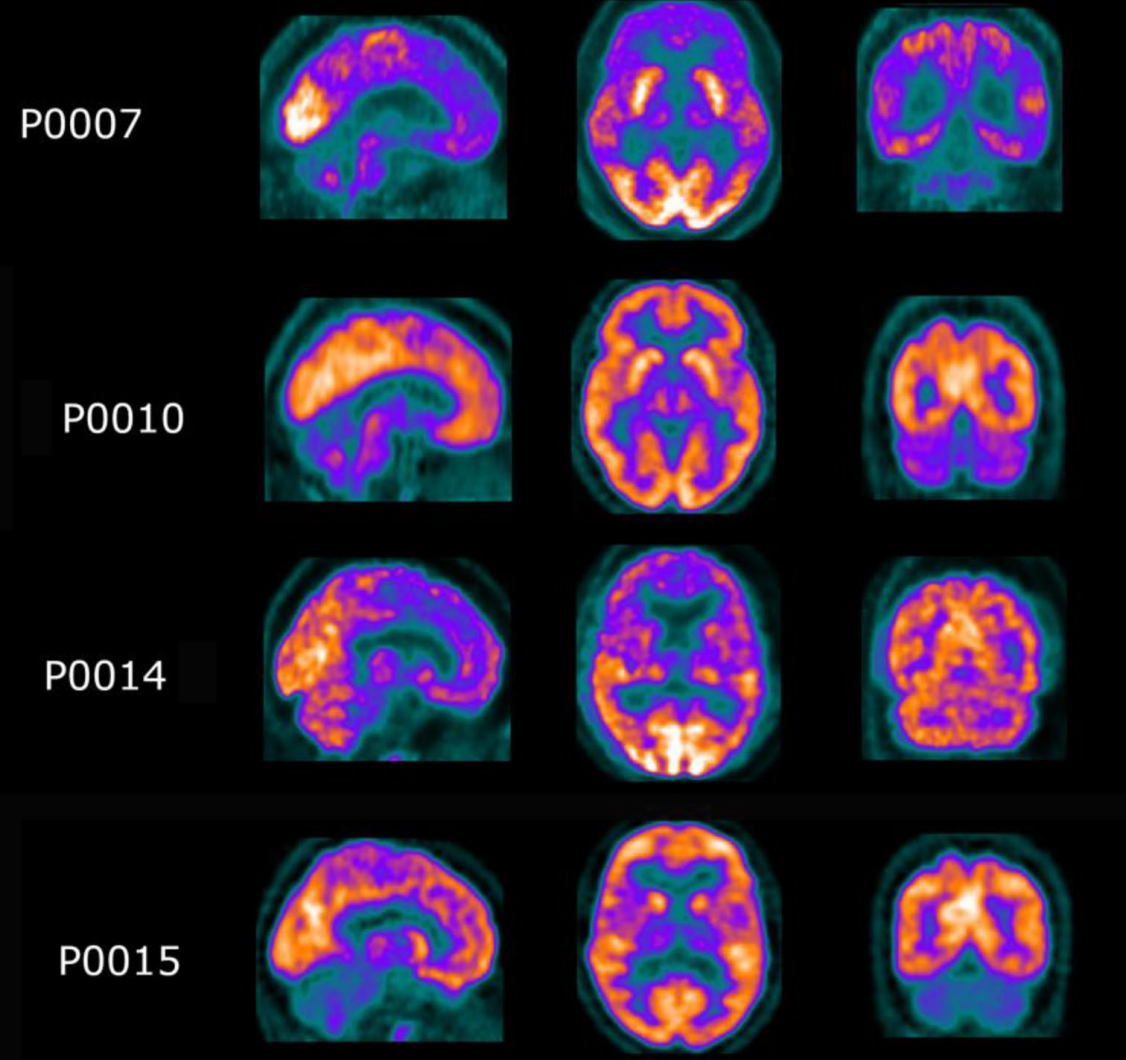
Representative slices of some of hypometabolism patterns observed during visual assessment

Figure 3 shows the results of the single-subject visual assessment of individual [^18^F]FDG PET images, while Fig. 4 shows representative slices of some of the observed patterns. The right-bottom corner of Fig. 3 shows the obtained median pattern, which is widely consistent with our group-level quantitative analyses. The most frequently reported visual findings were moderate to severe hypometabolism in the thalamus, cingulate and cerebellum. These three hallmarks were present in 17/22 patients, and at least two of the three were present in 21/22 patients. Hypometabolism in the basal ganglia was uncommon, and frontal metabolism was very common (17/22) but mostly reported as mild or moderate. Particularly, physicians reported severe frontal hypometabolism in a reduced number of patients, but it was not significantly identified in others. Parietal and occipital uptake were widely preserved in our cohort. About the usefulness of the derived group-level pattern, nuclear physicians described the correspondence between the patient’s image and the hypometabolism pattern as good or very good (2 or 3) for 13/22 (59%) patients. Supplementary Fig. 1 shows the individual quantitative patterns, which showed a good correlation with the visual evaluations. More details on the reporting of individual physicians can be found in Supplementary Table 4.

### Correlation between hypometabolism and atrophy patterns and clinical symptoms

Among clinical symptoms, ataxia was strongly associated with reduced cerebellum SUVR in region-based analysis (d = 1.88, *p* < 0.0001). This was confirmed in visual assessment, where 15/15 patients with a clinical presentation of ataxia exhibited moderate or severe cerebellar FDG hypometabolism. By contrast, only 2 out of 7 patients without ataxia symptoms showed cerebellar hypometabolism (1 mild, 1 moderate). A similar trend was observed for cerebellar atrophy (d = 0.87, *p* = 0.07). While no correlation was observed between any of the SUVR values and cognitive impairment, we did observe significantly lower GM_VOL values on the amygdala, inferior and middle temporal, and parahippocampus in cognitively impaired subjects (d > 1.09, *p* < 0.05). Regarding behavioural impairment, we did observe a relevant but non-significant association with lower SUVR values in several frontal regions, including the middle frontal gyrus and several orbitofrontal regions (d = 0.86, *p* < 0.07). In visual analysis, 7/14 patients of the patients presented behavioural impairment presented moderate or severe frontal hypometabolism, compared to 3 out unimpaired patients. Finally, we did not observe differences in SUVR between patients with and without miglustat treatment, but untreated patients showed a significantly lower hippocampal volume (d = 1.47, *p* < 0.01).

### Longitudinal changes in glucose metabolism

**Fig. 5:**
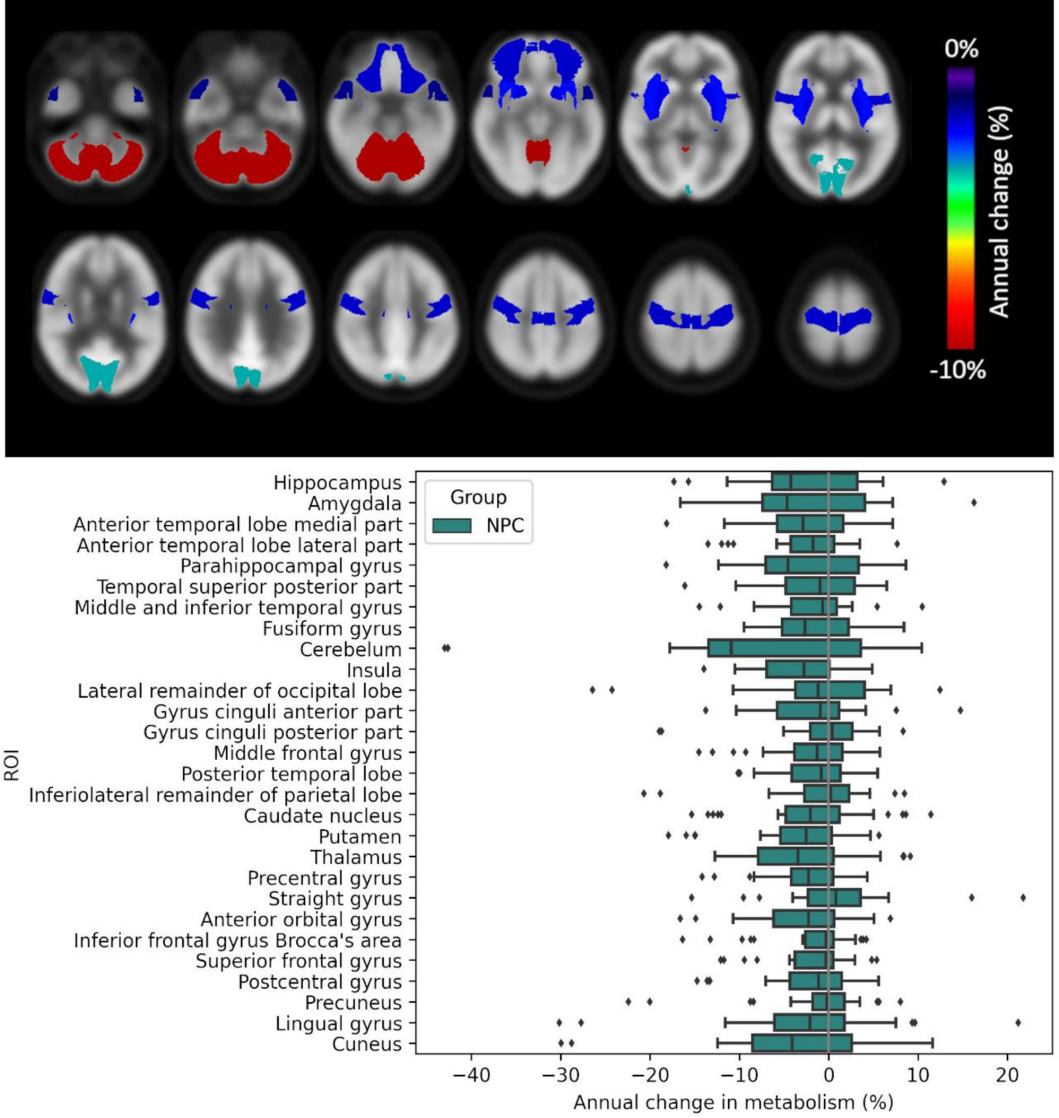
Average inter-annual change in regional glucose metabolism (up) and annual change in metabolism per region (down)

Figure 5 presents the longitudinal changes in brain metabolism averaged over all NP-C patients, showing percentage uptake reductions per year. Statistically significant decline in glucose metabolism was observed in the cerebellum (12%, *p* < 0.01), as well as to minor degrees in the insula, thalamus, putamen, precentral gyrus and anterior orbital gyrus (3–5%, *p* < 0.05). No region showed increases in metabolism or asymmetrical changes. Linear mixed effect models did not show a significant effect of ataxia or cognitive impairment at baseline on hypometabolism progression. Patients treated with miglustat showed a statistical trend towards slower progression of cerebellar hypometabolism compared to untreated patients (*p* = 0.10).

## Discussion

We present a cross-sectional and longitudinal structural MRI and [^18^F]FDG PET neuroimaging study in a multicentre cohort of twenty-two NP-C patients. Our baseline group-level analysis confirmed all expected MRI abnormalities summarized in the current recommendations for the detection and diagnosis of NP-C [19], and were consistent with previously described NP-C neuropathology [8, 9]. Regarding [^18^F]FDG PET, the derived hypometabolism pattern extended throughout the cerebellum, thalamus, midbrain, basal ganglia, cingulate cortex and large frontal, parietal and temporal bilateral regions. On the one hand, [^18^F]FDG PET showed to be more sensitive than MRI, providing higher effect sizes across all the affected areas (see Fig. 2). Furthermore, hypometabolism was also found in areas without atrophy, even though MRI scans were acquired a few months after the [^18^F]FDG PET scans on average (see Supplementary Table 2). Thus, our results support that PET may be able to detect earlier signs of neurodegeneration and provide higher effect sizes compared to MRI in NP-C. Based on these findings, [^18^F]FDG PET should be considered as a useful clinical tool in the diagnostic workup. However, while MRI abnormalities in NP-C are well-defined and fully aligned with our findings, the definition of a specific hypometabolism pattern associated with NP-C is still under debate. On this matter, our results provide additional evidence to refine the definition of an NP-C specific hypometabolism pattern [26–28]. According to our group-level analysis, NP-C is characterized by moderate/severe bilateral and symmetrical hypometabolism in the cerebellum, the thalamus, and the cingulate gyrus, with variable involvement of bilateral fronto-temporal regions. In contrast to previous works [25, 37], occipital metabolism was widely preserved in our cohort.

To refine the obtained pattern and to assess whether it could be useful in the diagnosis of individual NP-C patients, we performed single-subject visual and quantitative analysis. Two independent nuclear physicians evaluated the PET images, and degree of visually rated hypometabolism was defined by consensus. While visual ratings generally agreed well with the quantitative data, parietal changes were rarely identified in the visual ratings despite being observed in the quantitative group-level analysis (see Fig. 3). This discrepancy may be relevant for the elaboration of diagnostics guidelines, as visual assessment is still the primary method for evaluating brain [^18^F]FDG PET images in the clinic. In agreement with previous studies [25], frontal hypometabolism was present in most patients, but this was mostly rated as mild in the nuclear physician reports, where only 10/22 subjects had a rating of moderate or severe frontal hypometabolism. This led us to define cerebellar, thalamus and cingulate hypometabolism as the distinctive hallmark features of NP-C. These three hallmarks were present (rated as moderate or severe) in 17/22 patients, and at least two of the three were present in 21/22 patients. The two physicians reported that the derived group-level pattern was recognizable (good or very good correlation) in around 60% of the patients. 36% of the patients were considered to have an average correlation with the pattern, which suggests a high variability of [^18^F]FDG PET findings in NP-C. Interestingly, this variability in individual hypometabolism patterns was poorly correlated with that of clinical symptomatology. Noteworthy, a highly significant correlation (d = 1.88, *p* < 0.0001) was observed between ataxia and severe cerebellar hypometabolism. A smaller effect size, although statistically significant, was also observed between cerebellar atrophy and ataxia (d = 0.87, *p* = 0.07). These are expected finding, as ataxia in NP-C is known to have its pathological origin in the cerebellum [11]. The association between cortical neurodegeneration and cognitive-behavioral changes was more intricate, as none of the studied patients exhibited intact cortical metabolism. While we observed a significant correlation between frontal hypometabolism in quantitative analysis, with a small effect size (d = 0.86, *p* < 0.07), this correlation was not reproducible in visual ratings, suggesting that this effect size was not sufficient to translate into clinical relevance or may have limited practical significance. Similarly, cognitive impairment was associated with reduced grey matter volumes in several medial temporal lobe regions. Although, most of patients were cognitively unimpaired, these must be considered still as exploratory results. Larger sample sizes may be needed to draw reliable conclusions regarding the associations between neurodegeneration in different areas and cognitive/behavioural symptomatology in NP-C.

Finally, we performed longitudinal analysis, where we were able to measure notable decreases of metabolism in the cerebellum (annual decrease of 10–12%, *p* < 0.01) as well as a less significant, and less pronounced decline in the insula, thalamus, putamen, precentral gyrus, and anterior orbital gyrus (3–5%, *p* < 0.05). To date, longitudinal [^18^F]FDG PET had only been carried out in one cohort of sixteen paediatric patients, demonstrating that metabolic decline can be stabilized in children under treatment with miglustat [38]. Similarly, previous MRI-based studies could provide evidence for a slowing of cerebellar and subcortical atrophy by miglustat treatment [39]. In our adult-onset NP-C cohort, we also observed a statistical trend towards slower progression of cerebellar hypometabolism in treated patients. While this correlation was not significant, our results partially support these findings and suggest that inter-annual changes in cerebellar metabolism may be a viable candidate biomarker for assessing disease progression and treatment response in NP-C. It is important to note that some of these patients remain under treatment and continue to undergo longitudinal [18F]FDG PET scans. We anticipate that with an extended follow-up, further analyses may reveal more conclusive differences in the progression of brain hypometabolism between treated and untreated patients.

The current study also presents a series of limitations. First, our study was carried out in a reduced number of patients (*n* = 22). In this regard, it is important to consider that NP-C is a rare and highly disabling disease, and this is, to the best of our knowledge, still the largest [^18^F]FDG PET study of a NP-C cohort. Additionally, some concessions had to be made on the original protocols to include as many patients as possible. Thus, to ease operations at the collaborating centers, PET acquisitions for this project mostly used the same workflows used for the clinical PET acquisitions from each collaborating center. Although this is an evident limitation, it could be still considered methodologically acceptable since all protocols fall into the window of recommended protocols by the EANM guidelines. Furthermore, MRI imaging was delayed several months in some cases, and follow-up being only performed in a limited sample of twelve patients. On the other hand, longitudinal MRI acquisitions were not performed, and follow-up imaging limited to PET imaging to reduce patients’ strain, as most patients suffered significant discomfort during the MRI procedure at baseline. Consequently, our comparison between both modalities was limited to baseline data. Regarding the image analysis, another limitation was the age disparity between patients and controls. The included intensity normalization of PET images together with the inclusion of age as a covariate in our analyses should help us minimizing potential age-related effects. Future studies should consider using age-matched control groups to further validate these findings. Finally, clinical assessment has been reported mainly based on clinical descriptions, instead of using dedicated scales to assess the severity of variables such as ataxia or cognitive impairment, which would have allowed us to report correlations between clinical and imaging variables in a more accurate manner.

## Conclusions

Taken together, our cross-sectional and longitudinal findings delineate a discernible hypometabolism pattern specific to NP-C that distinguishes it from other neurodegenerative conditions. These changes on [^18^F]FDG PET largely exceeded changes seen in structural MRI, which is currently the best established imaging modality for aiding NP-C diagnosis. Within the broader pattern of hypometabolic brain regions in NP-C, individual [^18^F]FDG PET patterns showed considerable heterogeneity which was linked to clinical symptomatology and particularly the presence of ataxia. Longitudinally, the cerebellum exhibited accelerated neurodegeneration compared to other brain regions, albeit with a modest deceleration in patients undergoing miglustat treatment. Rates of change of cerebellar metabolism may constitute a promising imaging biomarker for tracking disease progression and evaluating treatment response in NP-C.

## Electronic supplementary material

Below is the link to the electronic supplementary material.

Supplementary Material 1

Supplementary Material 2

## Data Availability

The datasets generated during and/or analysed during the current study are available from the corresponding author on reasonable request.
