# Supplementary material for: Hypometabolism and atrophy patterns associated with Niemann-Pick type C
Source: EJNMMI Res. 2025 Feb 26;15:16. doi: 10.1186/s13550-025-01208-8 (PMC11865420; doi:10.1186/s13550-025-01208-8)
Supplement: Supplementary file 2 — Supplementary Material 2 [file 13550_2025_1208_MOESM2_ESM.docx]

| Scanner | Smoothing XY | Smoothing Z |
| --- | --- | --- |
| GE Advance Nxi | 4.0 | 3.0 |
| Siemens Biograph mMR | 6.0 | 5.5 |
| GE Discovery ST | 5.0 | 5.0 |
| GE Discovery 690 | 5.5 | 5.0 |

**Supplementary Table 1.** Differential smoothing applied by Neurocloud PET to each scanner.

| **Patient ID** | **Hospital** | **Sex** | **Age at baseline (years)** | **FDG-PET** | | | **MRI** |
| --- | --- | --- | --- | --- | --- | --- | --- |
|  |  |  |  | **n** | **Follow-up, (months)** | **Time from clinical onset (years)** | **Delay to FDG-PET (months)** |
| P0001 | Santiago | M | 41 | 2 | 11 | 28 | 0 |
| P0002 | Santiago | F | 33 | 2 | 20 | 3 | 0 |
| P0003 | Santiago | F | 65 | 2 | 15 | 6 | 0 |
| P0004 | Santiago | F | 29 | 1 | - | 14 | - |
| P0005 | Madrid | F | 53 | 1 | - | - | 0 |
| P0006 | Madrid | F | 55 | 1 | - | - | 0 |
| P0007 | Bellvitge | M | 17 | 3 | 43 | 11 | 9 |
| P0008 | Bellvitge | F | 35 | 1 | - | 19 | - |
| P0009 | Bellvitge | F | 14 | 3 | 39 | 0 | 8 |
| P0010 | Bellvitge | M | 28 | 5 | 71 | 12 | 47 |
| P0011 | Bellvitge | M | 26 | 5 | 83 | 9 | 35 |
| P0012 | Bellvitge | M | 47 | 3 | 29 | 13 | 26 |
| P0013 | Bellvitge | M | 58 | 1 | - | 26 | - |
| P0014 | Santiago | M | 50 | 1 | - | 7 | 0 |
| P0015 | Bilbao | M | 43 | 2 | 9 | 1 | 2 |
| P0016 | Bilbao | F | 41 | 2 | 9 | 26 | 5 |
| P0017 | Santiago | M | 17 | 2 | 96 | 4 | 0 |
| P0018 | Málaga | F | 38 | 1 | - | 15 | 0 |
| P0019 | Santiago | M | 26 | 2 | 32 | 7 | 0 |
| P0020 | Bellvitge | M | 24 | 1 | - | 12 | 12 |
| P0021 | Bellvitge | M | 44 | 1 | - | 30 | 14 |
| P0022 | Bellvitge | M | 43 | 1 | - | 24 | 8 |

**Supplementary Table 2.** Demographic characteristics and neuroimaging studies

| **Patient ID** | **Clinical assessment** | **NPC1 gene variants** | **Miglustat** |
| --- | --- | --- | --- |
| P0001 | **Ataxia**, psychosis, dystonia, vertical supranuclear gaze palsy and bulbar syndrome | p.[W942C] + p.[R1173G] | **Y** |
| P0002 | **Ataxia** and dysartrhya | p.[W942C] + p.[R1173G] | **Y** |
| P0003 | **Cognitive** and **behavioral** impairment, dystonia, vertical supranuclear gaze palsy, pseudobulbar palsy | *NPC2* (c.441+1G>A)*+? | **N** |
| P0004 | Dystonia and chorea, vertical supranuclear gaze palsy, pseudobulbar palsy | p.V562V (x2) | **Y** |
| P0005 | **Cognitive** and **behavioural** impairment, **ataxia**, bipolar disorder, vertical supranuclear gaze palsy, dystonia and bulbar syndrome | p.G1240R + p.R372W | **N** |
| P0006 | **Cognitive** and **behavioural** impairment, **ataxia**, vertical supranuclear gaze palsy,  bulbar syndrome | p.G1240R + p.R372W | **Y** |
| P0007 | **Cognitive** and **behavioural** impairment, **ataxia**, epilepsy, vertical supranuclear gaze palsy, dystonia and bulbar syndrome | p. [F995L] + p.[G992R] | **Y** |
| P0008 | **Cognitive** and **behavioural** impairment, **ataxia**, vertical supranuclear gaze palsy, dystonia and bulbar syndrome | p.[R372H] + p.[T1036K] | **N** |
| P0009 | **Cognitive** and **behavioural** impairment and vertical supranuclear gaze palsy | p.[P434L] + p.[M754K] | **N** |
| P0010 | **Cognitive** and **behavioural** impairment, **ataxia**, vertical supranuclear gaze palsy, dystonia and bulbar syndrome | p.[R372H] (x1) | **N** |
| P0011 | **Cognitive** and **behavioural** impairment, **ataxia**, vertical supranuclear gaze palsy, dystonia and bulbar syndrome | p.[R978C] + p.[F995L] | **N** |
| P0012 | **Cognitive** and **behavioural** impairment, **ataxia**, vertical supranuclear gaze palsy, dystonia and bulbar syndrome | p.[L1117fsX3]+ p.[F1224L] | **Y** |
| P0013 | **Cognitive** and **behavioural** impairment, **ataxia**, bipolar disorder, vertical supranuclear gaze palsy, dystonia and pseudobulbar syndrome | p.[Q775P] | **Y** |
| P0014 | **Cognitive** and **behavioral** impairment, tremor myoclonus | p.[P1007A] (x2) | **N** |
| P0015 | **Cognitive** impairment, **ataxia** and vertical supranuclear palsy | p. [D944N] + p. [A1174V] | **N** |
| P0016 | **Cognitive** impairment, **ataxia**, dystonia and vertical supranuclear palsy | p. [D944N] + p. [A1174V] | **N** |
| P0017 | **Cognitive** impairment, **ataxia**, epilepsy, vertical supranuclear gaze palsy and pseudobulbar syndrome | p. [M1142T ]+ p. [A750-G765del] | **Y** |
| P0018 | **Cognitive** and **behavioural** impairment, **ataxia**, vertical supranuclear gaze palsy, dystonia, myoclonic seizuresband bulbar syndrome | c.(1554-1009G>A) + p. [V1165M] | **Y** |
| P0019 | Bradykinesia and dystonia, antecollis, vertical supranuclear gaze palsy and pseudobulbar syndrome | N/A | **N** |
| P0020 | **Cognitive** and **behavioural** impairment, vertical supranuclear gaze palsy, dystonia and bulbar syndrome | p.[R518W] + p.[G992W] | **N** |
| P0021 | **Cognitive** and **behavioural** impairment, mioclonus, vertical supranuclear gaze palsy and bulbar syndrome | p.[L1117fsX3]+ p.[F1224L] | **Y** |
| P0022 | **Cognitive** impairment, **ataxia**, vertical supranuclear gaze palsy, dystonia and bulbar syndrome | p.[P434L] + p.[M754K] | **Y** |

**Supplementary Table 3.** Clinical assessment and NPC1 gene variants

| **Patient ID** | **Frontal** | **Temporal** | **Parietal** | **Occipital** | **Cingulate** | **Thalamus** | **Basal Ganglia** | **Cerebellum** | **Pattern Matching** |
| --- | --- | --- | --- | --- | --- | --- | --- | --- | --- |
| **P0001** | 2 | 2 | 0 | 0 | 2 | 3 | 0 | 3 | 3 |
| **P0002** | 0 | 1 | 0 | 0 | 3 | 3 | 1 | 3 | 1 |
| **P0003** | 3 | 2 | 0 | 0 | 2 | 2 | 1 | 0 | 1 |
| **P0004** | 3 | 1 | 2 | 1 | 3 | 3 | 0 | 3 | 3 |
| **P0005** | 2 | 2 | 1 | 0 | 1 | 0 | 0 | 3 | 2 |
| **P0006** | 1 | 2 | 0 | 1 | 1 | 1 | 0 | 2 | 2 |
| **P0007** | 3 | 3 | 3 | 0 | 3 | 3 | 0 | 3 | 2 |
| **P0008** | 3 | 2 | 2 | 0 | 3 | 3 | 0 | 3 | 3 |
| **P0009** | 0 | 1 | 0 | 0 | 2 | 0 | 0 | 1 | 1 |
| **P0010** | 1 | 1 | 0 | 0 | 1 | 2 | 0 | 3 | 2 |
| **P0011** | 2 | 2 | 0 | 0 | 1 | 3 | 1 | 3 | 3 |
| **P0012** | 0 | 2 | 1 | 0 | 2 | 3 | 1 | 3 | 1 |
| **P0013** | 1 | 0 | 0 | 0 | 1 | 0 | 0 | 1 | 2 |
| **P0014** | 2 | 1 | 0 | 0 | 1 | 1 | 1 | 0 | 1 |
| **P0015** | 0 | 1 | 0 | 1 | 2 | 2 | 0 | 3 | 2 |
| **P0016** | 0 | 1 | 0 | 1 | 2 | 2 | 0 | 3 | 2 |
| **P0017** | 1 | 0 | 0 | 0 | 3 | 3 | 0 | 2 | 3 |
| **P0018** | 3 | 2 | 1 | 0 | 3 | 3 | 0 | 3 | 3 |
| **P0019** | 2 | 0 | 0 | 0 | 1 | 1 | 1 | 0 | 1 |
| **P0020** | 1 | 0 | 0 | 0 | 2 | 3 | 0 | 1 | 2 |
| **P0021** | 1 | 0 | 2 | 1 | 2 | 0 | 1 | 1 | 2 |
| **P0022** | 1 | 3 | 0 | 0 | 1 | 2 | 1 | 2 | 2 |
| **Average** | 1.45 | 1.32 | 0.55 | 0.23 | 1,90 | 1.95 | 0.36 | 2.09 | 1.68 |
| **Median** | 1 | 1 | 0 | 0 | 2 | 3 | 0 | 3 | 2 |

**Supplementary Table 4.** Results of the visual analysis and qualitative evaluation with the derived hypometabolism pattern.


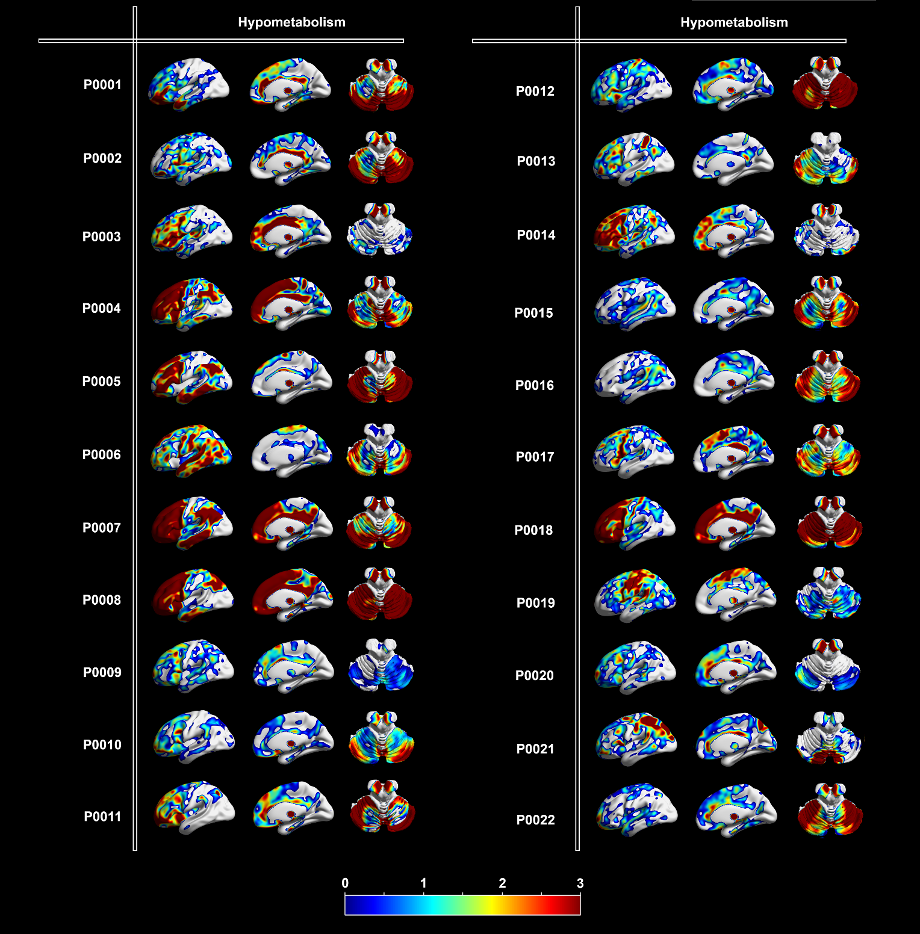


**Supplementary Figure 1:** Single-subject quantification patterns
