# Supplementary material for: Hypometabolism and atrophy patterns associated with Niemann-Pick type C
Source: EJNMMI Res. 2025 Feb 26;15:16. doi: 10.1186/s13550-025-01208-8 (PMC11865420; doi:10.1186/s13550-025-01208-8)
Supplement: Supplementary file 1 — Supplementary Material 1 [file 13550_2025_1208_MOESM1_ESM.docx]

First, we want to thank the editor and the reviewers. We highly appreciate the time they spent reviewing our manuscript and the provided feedback, which we believe has helped us to significantly improve the manuscript. We hope that the provided updated version will solve all the reviewers’ concerns. Below, we have provided detailed point-by-point responses to all the comments. The derived changes have been marked in red in the manuscript for a more comfortable evaluation of the revised manuscript.

**Reviewer #1:**

*The authors propose an original and interesting study on brain PET hypometabolism and MR atrophy in Niemann-Pick disease type C, a rare metabolic disorder.
They describe the neuroimaging profile at diagnosis, the clinical correlation with symptoms, and the follow-up after treatment.*

**Response:** We thank the Reviewer for the positive feedback and for providing specific suggestions for improvement, which we have addressed in detail below. In brief, we have incorporated the reviewer's suggestions regarding the PET and MRI quantification methodology, particularly concerning the age of the controls, issues related to intensity normalization, and finally we have added a new figure with the original FDG-PET images.

**Major comments:**

*1. The major limitation of the study is the age difference between patients and controls for PET imaging, and possibly also for MRI (age of controls for MRI is not disclosed). This strong limitation should be better taken in consideration in the discussion, and possibly also in the analyses.*

**Response:** All PET and MRI analysis in this manuscript considered the patient's age in the comparison with the controls, and, therefore, we feel that age differences between patients and controls may pose a limitation to our study. In this regard, we shall note that we have carried out several processing and analysis mitigations that should minimize the possible effects. Even so, we agree that it is a limitation that we should explicitly mention in the discussion. Below, some comments on this issue and the added text:

- With regards to PET, it is well-known that whole-brain FDG uptake decreases with age, however, the intensity normalization used in our PET image processing should in theory correct for these effects (see López-González et al. Neuroimage 2020, PMID: 32771619). In addition to global differences, several studies have identified age-related regional reductions in brain glucose metabolism, particularly in frontal regions (i.e. Loessner et al. JNM 1995, PMID: 7790936), including the anterior cingulate (Ishibashi et al. Sci Rep. 2018, PMID: 30068919). While our preprocessing cannot correct for these differences, the effect sizes reported in these studies are generally small compared to those observed in our research. Moreover, these reductions occur in the same direction of hypometabolism, suggesting that the differences we observed might be even more pronounced with an age-matched control group. Finally, all our voxel-wise analyses are corrected for age, which should also mitigate the contributions of age to the observed differences, if any. We have included a new sentence clearly stating this limitation to future readers in the revised manuscript (Discussion section):

*“Regarding the image analysis, another limitation was the age disparity between patients and controls. The included intensity normalization of PET images together with the inclusion of age as a covariate in our analyses should help us minimizing potential age-related effects. Future studies should consider using age-matched control groups to further validate these findings”*

- With regards to MRI, the Neurocloud volumetry software automatically selects the closest (best age-matched) subset of subjects (n=100) from the IXI and OASIS pooled database. While we don’t have the details of each of the age-matching selections, the following graph shows the age distribution of the IXI cohort (reproduced from Figure 4 of 10.1016/j.procs.2019.09.265). If this information is correct, we should assume that selected controls should be very closely age-matched. We have modified the corresponding section in the revised manuscript to clarify that the 100 control subjects were selected for each patient, and were not a fixed set (Statistical analysis section).

*“For structural MRI, the reference group included a large sample of 100 age-matched subjects from the IXI (brain-development.org) and OASIS (oasis-brains.org) databases, automatically selected by Neurocloud to best match the patient’s age.”*

*2. The diagnosis of neuroimaging in this context is unclear since diagnosis is now demonstrated by a genetic test.*

**Response:** Historically, diagnosing NPC has been a challenging process, relying on cholesterol esterification studies and filipin staining of cultured skin fibroblasts. Although genetic testing for NPC1 and NPC2 mutations is now widely available, approximately 10% of patients present only a single pathogenic mutation, with some cases revealing novel variants of uncertain significance. In such scenarios, the identification of innovative biomarkers capable of confirming the presence of NPC-like neurodegeneration could play a critical role in supporting the diagnosis. Additionally, several other conditions, including (but not limited to) spinocerebellar ataxias (types 2, 3, and 17), ataxia-telangiectasia, neuroacanthocytosis, Huntington’s disease, and dentato-rubro-pallido-luysian atrophy, are usually considered in the differential diagnosis before arriving at an NPC diagnosis. In this context, neuroimaging patterns associated with NPC may provide valuable insights to aid the differential diagnostic process. We have included a comment in the revised manuscript introduction expanding on these matters (Introduction section).

*“Like many rare disorders, diagnosis of NP-C is often challenging and the differential diagnosis against other conditions can be time-consuming, thus innovative biomarkers capable of confirming the presence of NPC-like neurodegeneration could play a critical role in supporting the diagnosis (6). Moreover, early diagnosis of NP-C is paramount, since a glucosylceramide synthase inhibitor treatment, miglustat, is available to slow down progressive neurological manifestations in adults and children. In this regard, biomarkers that can track disease progression are also needed (7).”*

*3. The methodology used for activity normalization should be discussed (since patients had diffuse cortical/subcortical impairment), in particular its possible application in clinical routine for visual interpretation.*

**Response:** Images were intensity normalized using a histogram-based data-driven method that calculates voxel-wise normalization maps between the image and the [18F]FDG template. The most prevalent value in the derived normalization map (i.e. the histogram peak) is selected as the global normalization factor. This method has been described and validated in detail in a previous publication (López-González et al. Neuroimage 2020, [PMID:](https://doi.org/10.1016/j.neuroimage.2020.117229) 32771619), demonstrating superior performance over conventional methods and robustness in cases with diffuse hypometabolism, such as NPC cases. Notably, this method is among those cited in the EANM procedure guidelines for brain PET imaging using [^18^F]FDG, version 3 (Guedj et al. EJNMMI 2022, PMID: 34882261). We have expanded the corresponding paragraph in the Methods section to provide additional information about the normalization method (Image processing section):

*“In brief, we calculated voxel-wise normalization maps by dividing the [18F]FDG template by the input image. Then, the most prevalent value in the derived normalization map (i.e. the histogram peak) is selected as the global normalization factor to scale the input image (34). After preprocessing, resulting PET images were smoothed with an additional 6x6x6 mm3 kernel prior to statistical analysis, resulting in a final intrinsic resolution of approximately 10 mm in every direction.”*

However, it’s important to note that the visual assessments presented here were conducted using the routing clinical workflow, thus physicians were allowed to adjust the image scale according to their preferences. Consequently, we cannot draw definitive conclusions about the impact of the described normalization method on visual interpretation in this context.

*4. It would be also nice to present individual PET slices showing these visual abnormalities (not only on statistical mapping)*

**Response:** We agree that the presentation of individual PET slices showing the expected visual abnormalities can be of great interest to clinicians. In the revised manuscript we have included a new Figure, which shows some of the expected patterns. First, P0007, with severe hypometabolism, P0010 and P0015 showing the most typical pattern with hypometabolism of the cerebellum, thalamus and anterior cingulate, but a relatively preserved cortex, and P0014 as an example of a patient with primary frontal involvement.

**Minor comments:**

*1. The study includes 22 patients. The cohort should not be described as (relatively) large as reported in the Abstract*

**Response:** Solved

**Reviewer #2:**

*Overall, this is an interesting paper that uses different methodological approaches to explore the relevance of FDG, as compared to volumetric MRI, in NP-C patients. There are, however, several aspects that should be considered before it might be suitable for its publication.*

**Response:** We thank the Reviewer for the positive feedback and for providing specific suggestions for improvement, which we have addressed in the point-by-point response below.

**Major comments:**

**Overall:**

*1. Please provide the effect size of the results. At this moment, much attention is given to the p-value, which is generally recommended to be used just as a threshold and not by the value itself, as it is of limited reproducibility.*

**Response:** We agree to change all results in the paper to show the effect size. We have made all the necessary changes to the text, figures, and tables. We have included Cohen’s d along with statistical significance for all the reported t-tests (i.e., p 11, lines 5-11), we have also updated Figure 2 so the scales are expressed in Cohen’s d, and we have modified the Methods section to report these changes.

**Methods:**

*2. Include the Standard Deviation of the injected dose for the FDG acquisitions.*

**Response:** After the Reviewer’s comment, we have noted an error in the reporting of the injected doses, as these were only reported for one of the centers. We have updated the Methods section to correctly report the injected doses. The range of injected activities is broad due to the wide range of body weights. For this reason, we consider it more appropriate to express the values within a minimum and maximum range:

*“after the intravenous injection of 185-370 MBq of [18F]FDG”.*

*3. Provide more details on the potential effect of the different acquisition protocols for FDG. For example, the combination of starting time 30 to 45 and acquisition 10 to 30 could provide very different acquisition windows.*

**Response:** We thank the Reviewer for highlighting this interesting point. To ease operations at the collaborating centers, PET acquisitions for this project mostly used the same workflows used for the clinical PET acquisitions from each collaborating center. This could be considered methodologically acceptable since all the reported intervals fall into the window recommended by the EANM (Guedj et al. EJNMMI 2022, PMID: 34882261) and the SNMMI (Arbizu et al. J Nucl Med 2024, PMID: 39419552) and also comply with the waiting times recommended by the used CE-marked quantification software (30-60 minutes). This reasoning is based on the following points:

- During this interval, the activity in the different brain regions is expected to reach a plateau, which is what allows static acquisitions and the calculation of ratios (i.e. applying intensity normalization), and we do not expect major differences due to the differences in waiting times.
- Similarly, about the acquisition times (and injected doses), each center used the standard acquisition times implemented in their clinical protocols. The different combinations of doses and acquisition times are usually fixed to provide good image quality, and they are expected both times and doses are expected to be reduced for modern scanners with much higher sensitivity (i.e. the Siemens mMR vs. the GE Advance NXi).

In any case, we acknowledge that this needs to be explicitly discussed in the paper, as it should be shown as a limitation. To make future readers aware of this concern, we have included the variability of acquisition protocols as a limitation in the Discussion section of the revised manuscript:

*To ease operations at the collaborating centers, PET acquisitions for this project mostly used the same workflows used for the clinical PET acquisitions from each collaborating center. Although this is an evident limitation, it could be still considered methodologically acceptable since all protocols fall into the window of recommended protocols by the EANM guidelines.*

*4. Provide more details on the harmonization kernels used between PET scanners (e.g., in supplementary material).*

**Response:** We thank the Reviewer for this insightful suggestion. We have included a new Table with the harmonization kernels provided by the software manufacturer on the Supplementary Material of the revised manuscript (Supplementary Table 1):

| Scanner | Smoothing XY | Smoothing Z |
| --- | --- | --- |
| GE Advance Nxi | 4.0 | 3.0 |
| Siemens Biograph mMR | 6.0 | 5.5 |
| GE Discovery ST | 5.0 | 5.0 |
| GE Discovery 690 | 5.5 | 5.0 |

**Supplementary Table 1.** Differential smoothing applied by Neurocloud PET to each of the used scanners.

Furthermore, Supplementary Table 1 was referenced in the Image processing Section as follows:

*PET pre-processing started with the application of scanner-specific smoothing kernels provided by Neurocloud for each scanner were applied, aiming to obtain a uniform isotropic resolution of 8 mm FWHM (Supplementary Table 1).*

 *5. Were the reference subjects matched also by sex?*

**Response:** Reference subjects were not matched only by age, but our cohort is not over-representing any sex (41% woman) and the healthy databases are approximately balanced. In any case, we have included sex as a covariate in most of the presented analyses.

*6. (Pg7 Ln25) As mentioned above, the acquisition window is broad. Please provide more details on the one used for the reference group.*

**Response:** The reference group was acquired at our center using the GE Advance NXi using the same acquisition protocol used for this study. Acquisitions were performed after an intravenous injection of 370 MBq of [^18^F]FDG. Patients were asked to lay at rest in a dark and quiet room. Starting 45 minutes after injection, the patients were scanned during 30 minutes for emission data and 15 minutes for transmission data, required for attenuation correction. The imaging device was a whole-body PET scanner (GE Advance NXi). All images were reconstructed with an iterative method using 4 iterations and 16 subsets. This information was incorporated into the revised manuscript (Statistical analysis section):

*Briefly, subjects were scanned in a GE Advance NXi for 30 minutes, starting 45 minutes after the injection of 370 MBq of [18F]FDG. Images were reconstructed with an iterative reconstruction method using 4 iterations and 16 subsets.*

*7. Provide the final voxel size for MRI and PET images used for the analysis. Moreover, assuming different voxel sizes between modalities, what is the rationale for using the same number of voxels to threshold the results (cluster size)?*

**Response:** The output voxel size for pre-processed images is the same for both modalities: 1.5x1.5x1.5 mm^3^. This is the default output voxel size for CAT12, and it was adapted by Neurocloud for both MRI and PET processing. This information was included in the revised manuscript (Image processing section).

*“The output voxel size of the normalized images was 1.5x1.5x1.5 mm3.”*

8. Provide more details on the settings used for the SPM voxel-based analysis (e.g., extra smoothing, scaling, mask)

**Response:** Despite our group-wise analysis was performed mainly following the defaults proposed by CAT12, we have included additional information in the revised manuscript, such as information about the smoothing applied prior to SPM analysis (Image processing Section). We also included information about the mask used for the analysis, which was a generic parenchyma mask provided by CAT12 for both MRI and PET (Statistical Analysis Section). Finally, about the scaling, we used a histogram-based data-driven method. This method has been described and validated in detail in a previous publication (López-González et al. Neuroimage 2020, [PMID:](https://doi.org/10.1016/j.neuroimage.2020.117229) 32771619), demonstrating superior performance over conventional methods and robustness in cases with diffuse hypometabolism, such as NPC cases. This method is among those cited in the EANM procedure guidelines for brain PET imaging using [^18^F]FDG, version 3 (Guedj et al. EJNMMI 2022, PMID: 34882261). We have expanded the corresponding paragraph in the Image Processing section to provide additional information about the scaling method. Below, all added paragraphs:

*“Segmented grey matter (GM) tissue maps were smoothed using an isotropic 10x10x10 mm3 kernel to remove differences related to inter-individual differences prior to statistical analysis.”*

*“PET pre-processing started with the application of scanner-specific smoothing kernels provided by Neurocloud for each scanner were applied, aiming to obtain a uniform isotropic resolution of 8 mm FWHM (Supplementary Table 1).”*

*“In brief, we calculated voxel-wise normalization maps by dividing the [18F]FDG template by the input image. Then, the most prevalent value in the derived normalization map (i.e. the histogram peak) is selected as the global normalization factor to scale the input image (34). After preprocessing, resulting PET images were smoothed with an additional 6x6x6 mm3 kernel prior to statistical analysis, resulting in a final intrinsic resolution of approximately 10 mm in every direction.”*

*9. The voxel-based analysis of volumes was analyzed as "one-tailed" (Pg7 Ln21). Was the VOI-based analysis also performed one tail or two-tailed?*

**Response:** We thank the Reviewer for raising this point, which could indeed be confusing for future readers. To clarify, the performed voxel-based analyses were two-tailed, as it can be typically expected from SPM analysis. However, we focused our reporting on reductions in metabolism, while increases (i.e., hypermetabolism) were reviewed solely for quality control purposes. To simplify the manuscript and avoid potential misinterpretation, we have removed the following sentence: “Only reductions in volume were considered, while volume increases were only visually inspected to identify any potential artifacts.”. Of note, VOI-based analyses were also two-tailed.

10. Please add more details on the p-value used to consider statistical significance, the use (or not) of corrections for multiple comparisons, and the software packages used for statistical analysis.

**Response:** We have included information about the statistical thresholds used in the Statistical analysis section of the revised manuscript.

*Differences are reported as effect size (Cohen’s d) and statistical significance. Differences were considered significant at a p<0.01 (uncorrected).*

**Results:**

11. Move Tables 1 and 2 to supplementary material and replace them with plots summarizing the data.

**Response:** Table 1 and 2 were moved to Supplementary Table 2 and Table 3. In addition, a new Figure was created summarizing the clinical data from Table 2. We completely agree with the reviewer that this new figure allows for a clearer and simpler understanding of the clinical symptoms in our cohort.

**Figure 1.** Summary of clinical assessment

12. It is mentioned in the feet of Table 2 that subject P0019 had no NPC mutation. Therefore, according to inclusion criteria (Pg5 Ln29), this subject should be removed from the analysis.

**Response:** We understand the reviewer's concern regarding the inclusion of a patient without a confirmed genetic mutation. The final inclusion of this patient was a decision that was extensively discussed with the clinicians and geneticists of the team, before conducting the neuroimaging study. We concluded that the inclusion of this patient could be well justified based on two critical factors that we outline below:

- His clinical profile, the patient exhibits a highly suggestive clinical profile consistent with NPC, including bradykinesia, dystonia, antecollis, vertical supranuclear gaze palsy, and pseudobulbar syndrome. Among these, vertical supranuclear gaze palsy is considered a highly specific clinical hallmark of NPC, and its presence was a decisive factor in our decision to include this patient in the study. The constellation of these clinical features aligns closely with the well-established phenotypic spectrum of NPC, supporting a strong clinical suspicion of the disease.
- Genetic testing has inherent limitations, since the gene panel performed in this case did not cover intronic or regulatory regions, where causal variants may reside. Additionally, certain genetic variants in the NPC1 and NPC2 genes are known to be difficult to detect with standard genetic panels. Consequently, the absence of identified mutations does not definitively rule out the diagnosis of NPC, particularly in a patient presenting with such a characteristic clinical phenotype.

We acknowledge that the lack of a confirmed genetic mutation represents a limitation, and we have explicitly addressed this point in the discussion section of the manuscript. However, we believe that excluding this patient solely based on the absence of a detected mutation would risk overlooking a potentially valuable clinical case that contributes to a better understanding of the clinical spectrum of NPC. Furthermore, excluding this patient from the neuroimaging study would not have any impact on the overall results.

Next paragraph has been added to the revised version of the paper:

Patient Recruitment and Clinical Evaluation:

*“Eligible subjects must have a genetically confirmed diagnosis of NP-C disease (any combination of clinical manifestations was permitted) or inconclusive genetic diagnosis combined with characteristic NP-C symptomatology (evaluated by a panel of neurologists specialized in NPC).”*

13. In Figure 1 B, please provide the color code based on the effect size, not the p-value.

**Response:** As mentioned in response to comment 1, we updated Figure 1 (now Figure 2 in the revised paper), so the scales are expressed in Cohen’s d (both A and B panels).

14. Please add more details on the correlation between the FDG and VOL values and consider further analyses for head-to-head comparisons of methods and the potential superiority of FDG over MRI. Also, provide effect sizes and some plots on their relations with the clinical symptoms.

**Response:** We thank the Reviewer for this insightful suggestion. While the Results in Figure 1 (now Figure 2 in the revised paper) clearly point to a correlation between atrophy and hypometabolism, providing quantitative metrics is a nice addition to the manuscript. In brief, we have used regional VOI-based values and the Pearson’s r to assess the correlation, and we observed a significant positive correlation between atrophy and hypometabolism on several relevant areas, such as the cerebellum, thalamus, posterior cingulate, fusiform gyrus, and middle and inferior frontal gyrus (r>0.46, p<0.05). This information has been added to the revised manuscript (Section: Correlation between hypometabolism and atrophy patterns and clinical symptoms).

**Discussion:**

15. Overall, the discussion of the results in this section will be better supported when measures of effect sizes are reported in the body of the text, moving beyond the p-value alone. For example, the bigger effect size of FDG above MRI is supported only by the visual inspection of Figure 1.

**Response:** Effect sizes were incorporated into the numbers referenced in the Discussion section. In this way, a stronger correlation between FDG and ataxia symptoms is clearly demonstrated compared to MRI, both in terms of effect size and p-value:

*“Noteworthy, a highly significant correlation (d=1.88, p<0.0001) was observed between ataxia and severe cerebellar hypometabolism. A smaller effect size, although statistically significant, was also observed between cerebellar atrophy and ataxia (d=0.87, p = 0.07).”*

Furthermore, effect size values explain the inconsistency between visual and quantitative analyses regarding the hypometabolism in the frontal cortex:

*“While we observed a significant correlation between frontal hypometabolism in quantitative analysis, with a small effect size (d=0.86, p<0.07), this correlation was not reproducible in visual ratings, suggesting that this effect size was not sufficient to translate into clinical relevance or may have limited practical significance.”*

16. Expand the discussion on the results found on miglustat in the current dataset.

We did not include a detailed discussion on the impact of miglustat treatment because no statistically significant differences were found between the treated and untreated groups in terms of hypometabolism in the cerebellum (or other regions). While a trend towards slower progression was observed in patients treated with miglustat (p=0.10), the current sample size and follow-up period may not have been sufficient to detect a significant effect. It is important to note that some of these patients remain under treatment and continue to undergo longitudinal [18F]FDG PET scans. We anticipate that with an extended follow-up, further analyses may reveal more conclusive differences in the progression of brain metabolism between treated and untreated patients. This paragraph was included in the Discussion section:

*“It is important to note that some of these patients remain under treatment and continue to undergo longitudinal [18F]FDG PET scans. We anticipate that with an extended follow-up, further analyses may reveal more conclusive differences in the progression of brain hypometabolism between treated and untreated patients.”*

17. Please consider the need (or not) for partial volume correction methods in FDG.

**Response:** Considering partial volume correction in this study seems irrelevant, as the hypometabolic regions are extensive, without small or focal areas that could be significantly affected by partial volume effects. Additionally, there are no off-target regions in FDG-PET imaging, unlike other radiotracers such as amyloid and tau PET, where these off-target regions could potentially contaminate the measurements in the target regions due to spill-in counts.
